# Effects of the Mixing Protocol on the Self-Assembling Process of Water Soluble Porphyrins

**DOI:** 10.3390/ijms22020797

**Published:** 2021-01-14

**Authors:** Maria Angela Castriciano, Sergio Cardillo, Roberto Zagami, Mariachiara Trapani, Andrea Romeo, Luigi Monsù Scolaro

**Affiliations:** 1 CNR—ISMN Istituto per lo Studio dei Materiali Nanostrutturati c/o Dipartimento di Scienze Chimiche, Biologiche, Farmaceutiche ed Ambientali, University of Messina, V.le F. Stagno D’Alcontres, 31-98166 Messina, Italy; maria.castriciano@cnr.it (M.A.C.); mariachiara.trapani@cnr.it (M.T.); 2 Dipartimento di Scienze Chimiche, Biologiche, Farmaceutiche ed Ambientali, University of Messina and C.I.R.C.M.S.B V.le F. Stagno D’Alcontres, 31-98166 Messina, Italy; cardillo.sergio94@gmail.com (S.C.); rzagami@unime.it (R.Z.)

**Keywords:** porphyrins, J-aggregates, aggregation kinetics, symmetry breaking, chiral supramolecular assemblies

## Abstract

The hierarchical self-assembling kinetics of the porphyrin 5,10,15,20-*tetrakis*(4-sulfonatophenyl)porphyrin (H_2_TPPS_4_^4−^) into J-aggregates at high ionic strength under acidic conditions and eventually in the presence of an added chiral templating agent (tartrate) were investigated through UV/Vis spectroscopy, resonance light scattering, and circular dichroism (CD). The effect of changing the mixing order of the various components in the solution on the kinetic parameters and the expression of chirality on the final J-aggregates was evaluated. In this latter case, only when the chiral tartrate anion is premixed with the porphyrin, the resulting nano-architectures exhibit CD spectra that reflect the handedness of the chiral inducer. We discuss a general mechanistic scheme, with the involvement of ion pairs or dimers that offer an alternative pathway to the aggregation process.

## 1. Introduction

**Scheme 1:**
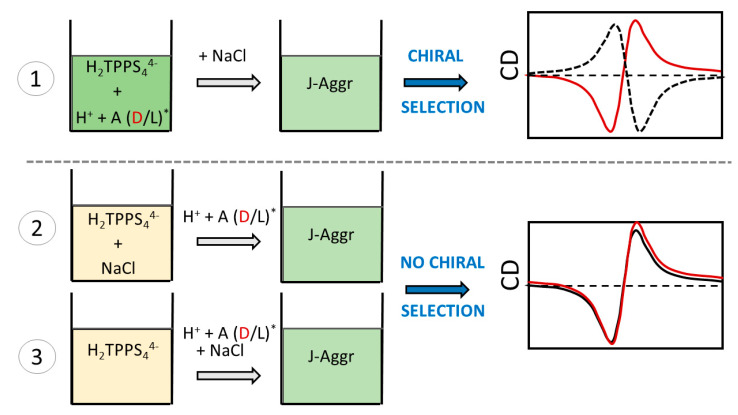
Mixing protocols adopted for the J-aggregation of the H_2_TPPS_4_^4−^ porphyrin (see text) with the role of D- and L- enantiomers of tartrate anion in the chiral selection.

Aggregation of porphyrins is a general phenomenon occurring in this class of compounds and is mainly ascribable to different intermolecular forces acting among the basic monomeric units [1]. The electronic spectra of these species are largely affected by the relative structural arrangement and electronic coupling of the chromophores within the aggregates. The exciton model developed by Kasha predicts two limiting cases for an edge-to-edge and a face-to-face disposition, leading to a shift of the relevant electronic transitions to lower (J-type) or to higher energies (H-type), respectively [2]. In this context, J-aggregates of porphyrins have been largely investigated due to their peculiar electronic and optical properties [3,4,5,6,7,8,9,10,11,12,13,14]. Sulphonato-phenyl-porphyrins and, in particular, the tetra-anionic 5,10,15,20-*tetrakis*(4-sulfonatophenyl)porphyrin (H_2_TPPS_4_^4−^) have received a lot of attention since they are soluble in water, and aggregation can be triggered by a proper choice of experimental conditions (concentration, pH, temperature, ionic strength, etc.) [5,15,16,17,18,19,20,21,22,23,24,25,26,27,28,29,30,31]. Upon protonation, the diacid form H_4_TPPS_4_^2−^, a dianion, initiates a self-assembling process mediated by electrostatic hydrogen bonding and solvophobic interactions. An intriguing feature in some of these aggregated species is the emergence of chirality, either imprinted through chemical [32,33,34,35,36,37,38,39,40,41,42,43] or physical bias [44,45,46,47,48,49,50,51,52] or deriving from spontaneous symmetry breaking [27,53,54]. Anyway, diverse results of the observed morphology, sizes, kinetics, and chirality of aggregates obtained under a variety of experimental conditions have focused attention on the importance of the mixing protocols adopted in the different investigations [23,27,55]. In previous studies we demonstrated that in a simple acid triggered aggregation, if the acid is added to a pre-diluted porphyrin solution (referred as porphyrin first (PF) protocol), the kinetics evolve through a slow nucleation phase or lag-time, followed by a quite fast autocatalytic growth [27]. The resulting J-aggregates exhibit circular dichroism (CD) as the spectral signature of an enantiomeric imbalance in the population of the nano-assemblies in solution. On the contrary, when a porphyrin stock solution is added to an acid solution (referred as porphyrin last (PL) protocol), the initial induction period is absent in the kinetic profiles that assume a faster stretched exponential form while the final CD spectra are silent. When the pH is in the range 2–4, self-aggregation of H_4_TPPS_4_^2−^ is also fostered by increasing ionic strength, e.g., adding sodium chloride [18,24] or polyamines [20,56,57]. On changing the mixing order, the difference in kinetic behavior is so striking that a sort of “YES/NO” effect has been pointed out in literature. This occurs especially at low porphyrin concentration, where the concentration effect impacts the rates, slowing down dramatically the aggregation process [23,56]. Indeed, this effect has been fruitfully exploited to obtain a hierarchical control on the supramolecular assembling process, selecting from different aggregation pathways [23,58,59]. Anyway, the addition of a third ingredient, i.e., the salt, to the mixing procedure increases the number of different ways to mix up the reagents. Furthermore, when chirality is imprinted in J-aggregates through a chemical inducer, this fourth latter component requires a precise timing for its involvement in the hierarchical path to the complex system. In previous investigations, we showed that using a PL protocol and changing the concentration of porphyrin and the overall ionic strength, different mesoscopic structures were obtained, i.e., fractal clusters at low porphyrin concentration and high ionic strength, and rod-like aggregates at higher porphyrin concentration and lower ionic strength. In both regimes, chiral selection has been detected as consequence of the handedness of the inducer (tartrate), even if the sign of the observed CD spectra for the same enantiomeric form of the chiral anion was reversed by changing the overall structural arrangement [24]. The same kind of chiral control was detected for J-aggregates obtained in confined environments, i.e., in the water-pool of micro-emulsions [34]. Experiments that are more recent revealed a strong kinetic discrimination between L and D-tartrate, when adopting PF mixing protocols under quite acidic conditions, but with induced CD bands reversed with respect to the initial PL experiments [35]. Therefore, the diverse experimental evidence suggests that in addition to the concentration of porphyrin, pH, and ionic strength, the mixing protocol plays an important role in controlling the eventual handedness of chiral J-aggregates. Here we report on a comparative kinetic analysis of the formation of J-aggregates starting from the H_2_TPPS_4_^4−^ porphyrin at selected acidic conditions in the presence of salt. In separate experiments, chiral tartrate will be also added to the system in order to check the impact of the different mixing protocols on the expression of chirality. We anticipate that only when the chiral species is premixed with the porphyrin will the handedness of the chiral inducer be transferred to the final aggregate (see Scheme 1).

## 2. Results and Discussion

**Figure 1:**
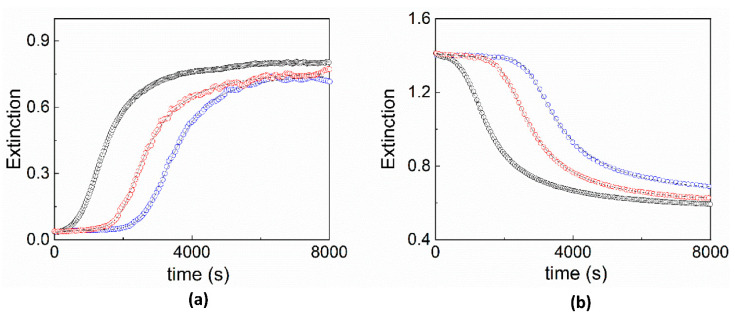
Extinction kinetic profiles recorded at 492 nm (**a**) and 434 nm (**b**) for the aggregation of H_2_TPPS_4_^4−^ induced by adopting three different reagent mixing order protocols (black traces—mix 1; red traces—mix 2; blue traces—mix 3). Experimental conditions: [H_2_TPPS_4_^4−^] = 3 μM; [HCl] = 0.3 M; [NaCl] = 0.2 M, T = 298 K. The solid lines show the best fit of the experimental data to Equation (1).

The UV/vis absorption spectra of H_2_TPPS_4_^4−^ porphyrin are deeply influenced by the protonation and aggregation state. Under neutral pH, the porphyrin is a tetra-anion H_2_TPPS_4_^4−^ and exhibits a very intense B-band at 414 nm (ε ≈ 5 × 10^5^ M^−1^ cm^−1^), accompanied by four Q bands of much lower intensity at longer wavelength. The central nitrogen atoms can be easily protonated in two very close steps (pK_a_ ≈ 5) [60] and, consequently, below pH 4 the porphyrin is in its diacid H_4_TPPS_4_^2−^ form, characterized by an intense B-band at 434 nm, while the Q-region shows only two bands. Upon aggregation under acidic conditions, the presence of a rather sharp and intense peak at 492 nm (J-component), together with a less intense one at 422 nm (H-component) and a set of Q-bands in the red portion of the spectra are the spectroscopic features of J-aggregates [4,13,61]. In order to investigate the role of the mixing order in the presence of salt, we decided to adopt a PF protocol, since the PL one would lead to much faster aggregation rates without a clear nucleation phase [27]. Therefore, we selected three different protocols: (i) adding HCl to a pre-diluted porphyrin solution, and subsequently adding NaCl (mix 1); (ii) adding NaCl to a pre-diluted porphyrin solution and then the acid (mix 2); (iii) adding a premixed solution of NaCl and HCl to a pre-diluted porphyrin solution (mix 3) (for details, see the Experimental Section). Figure 1 shows a comparison of the kinetic traces for the changes in the extinction of the various experiments collected at 434 and 492 nm, ascribable to the extinction maxima of diacid and J-aggregates species, respectively. All the kinetic profiles have a sigmoidal behavior, with quite different lag phases or induction times. It is rather evident from kinetic traces relative to J-aggregates growth that mixing protocol 2 and 3 lead to a certain degree of instability in the final structures, probably due to the formation of very large clusters that tend to precipitate at longer times.

The kinetic analysis of these data was fulfilled using a well-established model (see Equation (1) below, developed by Pasternack et al. [62,63]. Based on the auto-catalytic growth via a *k_c_* pathway, the model starts from an initial seed containing *m* porphyrin units. The self-assembling proceeds through a surface-type reaction dominated by a power law with a typical exponent *n*. A non-catalyzed pathway was also considered with the rate constant *k*_0_. All the relevant kinetic parameters are collected in Table 1.

**Figure 2:**
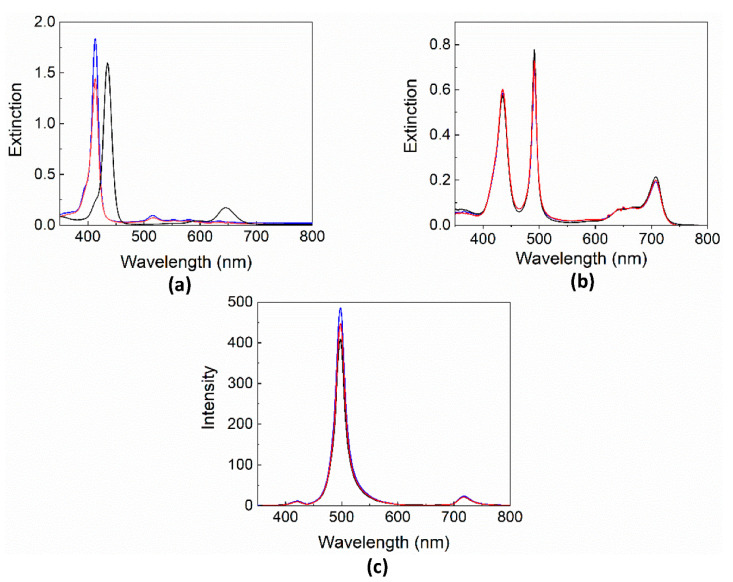
Extinction spectra for the initial species (**a**) and for the samples at the end of the aggregation (**b**) together with their corresponding resonance light scattering (RLS) (**c**) profiles by adopting three different reagent mixing order protocols (black traces—mix 1; red traces—mix 2; blue traces—mix 3). Experimental conditions: [H_2_TPPS_4_^4−^] = 3 μM; [HCl] = 0.3 M; [NaCl] = 0.2 M, T = 298 K.

For the sake of comparison, the kinetics of aggregation of H_2_TPPS_4_^4−^ triggered by HCl using a PF protocol have been also analyzed and reported [27]. Mixing protocol 1 evidences a striking difference in rate constant and in the lag-time when compared to the other two methods, leading to a higher value for *k_c_* and a shorter induction period (related to smaller values of the power exponent *n*). Figure 2 displays a comparison of the extinction spectra for the initial species and the eventual J-aggregates, together with their corresponding resonance light scattering (RLS) profiles, obtained by using the three different mixing protocols. Whatever the mixing protocol is, J-aggregates of H_2_TPPS_4_^4−^ display the same spectral features at the end of the self-assembling process, as proved by the slight differences among the various extinction (Figure 2b) and RLS (Figure 2c) spectra. On the contrary, extinction spectra acquired before triggering the aggregation process are quite different since the starting species depends on the initial addition in the mixing protocol (Figure 2a). In mix 1, the diacid porphyrin H_2_TPPS_4_^2−^ is immediately formed upon addition of acid (black line). In mix 2 and 3, the starting species is the H_2_TPPS_4_^4−^ porphyrin (414 nm, blue line) that immediately undergoes hypochromicity and a slight hypsochromic shift to 412 nm, upon salt addition (mix 2).

**Figure 3:**
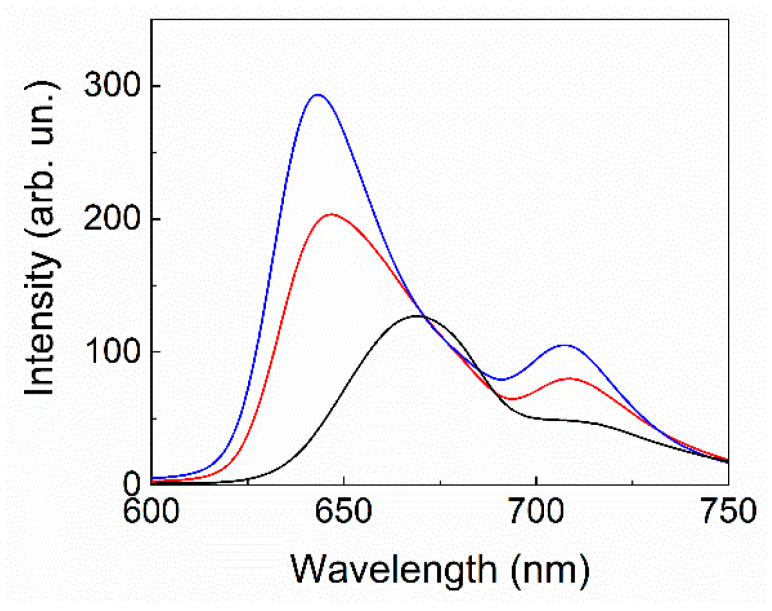
Fluorescence emission spectra comparison among the initial H_2_TPPS_4_^4−^ (blue trace), after the addition of NaCl (red trace) and the H_4_TPPS_4_^2−^ (black trace). Experimental conditions: [H_2_TPPS_4_^4−^] = 3 μM; [HCl] = 0.3 M; [NaCl] = 0.2 M; λ_ex_ = 420, T = 298 K.

Figure 3 shows a comparison of the fluorescence emission spectra among the initial H_2_TPPS_4_^4−^ porphyrin (blue trace), after the addition of salt (red trace) and the diacid porphyrin H_4_TPPS_4_^2−^ (black trace). All samples display the typical profile with two emission bands in the region 600–800 nm. As far as the H_2_TPPS_4_^4−^ porphyrin in the presence of salt is concerned, a partial quenching of the emission intensity, together with a shift to lower energies (Δλ = +6 nm), is detectable (red trace) with respect to the starting H_2_TPPS_4_^4−^ (blue trace). Fluorescence lifetime measurements were performed on these latter two samples. In agreement with its monomeric nature and in line with the literature [64], a mono-exponential decay is evident for the starting porphyrin with τ = 10 ns. Soon after adding NaCl 0.2 M, a bi-exponential decay with lifetime values τ_1_ = 10 ns (97%) and τ_2_ = 5 ns (3%) was measured. The percentage distribution of these values evolves with time, and after 40 min reaches an equilibrium situation of 90% for the longer and 10% for the shorter lifetime. This spectroscopic evidence suggests the formation of an H-type dimer of the tetra-anionic porphyrin and/or some ion pair with the sodium cations [64].

**Scheme 2:**
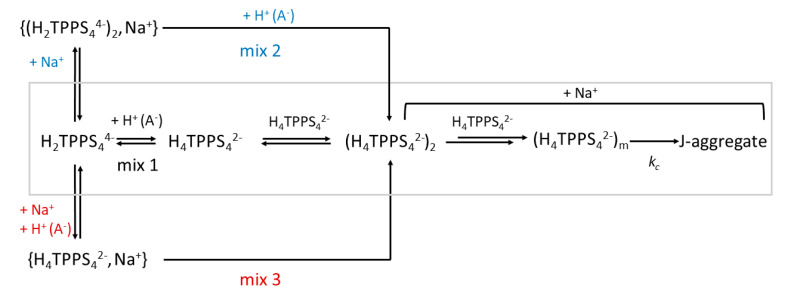
Mechanistic scheme proposed for the self-assembling process of H_2_TPPS_4_^4−^ porphyrin leading to J-aggregates, following the mixing protocols explained in the main text: (i) mix 1, addition of acid/chiral inducer followed by salt, (ii) mix 2, addition of salt followed by acid/chiral inducer, and (iii) addition of a premixed solution of acid/chiral inducer and salt to a pre-diluted porphyrin solution.

On the basis of the experimental findings, a general mechanistic scheme can be proposed (Scheme 2). In mix 1, after the initial fast protonation of H_2_TPPS_4_^4−^ to the diacid porphyrin H_4_TPPS_4_^2−^, the interaction between this monomeric species leads to a dimer (H_4_TPPS_4_^2−^)_2_ that is stabilized by electrostatic and solvophobic interactions. This species forms quite rapidly, even if in low concentration, and the subsequent uptake of other porphyrins originates the initial nucleus containing *m* units in the rate determining step of the entire process. From this event, the J-aggregates build up, following an exponential growth (via *k_c_*) consuming the monomeric units. The delayed addition of Na^+^ plays a role in the stabilization of the oligomeric species and the final J-aggregates. In mix 2 the addition of salt to H_2_TPPS_4_^4−^ promotes the quite slow formation of the H-type dimer with ion pairing of Na^+^, {(H_2_TPPS_4_^4−^)_2_, Na^+^}. This species is rapidly converted into (H_4_TPPS_4_^2−^)_2_ that leads to J-aggregates through the previously described path. When Na^+^ and the acid are simultaneously added to H_2_TPPS_4_^4−^, the formation of the H-dimer is largely suppressed and an ion pair between the diacid porphyrin and sodium cations {H_4_TPPS_4_^2−^, Na^+^} forms, which slowly interconverts into the dimer (H_4_TPPS_4_^2−^)_2_. Even if we have no spectroscopic evidence for this species, the much larger lag-time for the relative kinetic profiles (Figure 1, blue traces) suggests the slower extrusion or rearrangement of the sodium cations to allow for the interaction between the porphyrin units.

**Figure 4:**
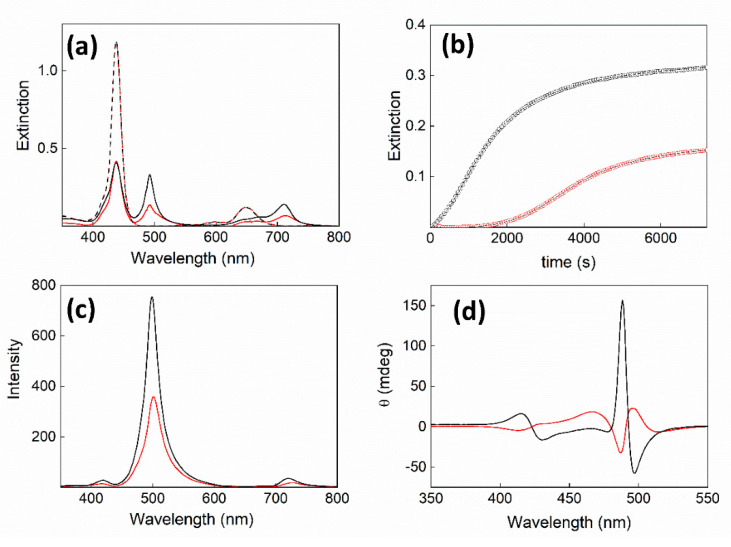
(**a**) Extinction spectra soon after initial mixing (dashed lines) and at the end of the aggregation (solid lines), (**b**) kinetic extinction time traces at λ = 492 nm (solid lines show the best fit of the experimental data according to Equation (1)), (**c**) RLS and (**d**) Circular dichroism (CD) spectra for the supramolecular assembling of H_2_TPPS_4_^4−^ induced by salt and tartrate buffer (L: black lines; D: red lines) using mix 1 protocol. Experimental conditions: [H_2_TPPS_4_^4−^] = 3 μM; [NaCl] = 1.5 M; tartrate buffer 100 mM, pH 2.7, T = 298 K.

**Figure 5:**
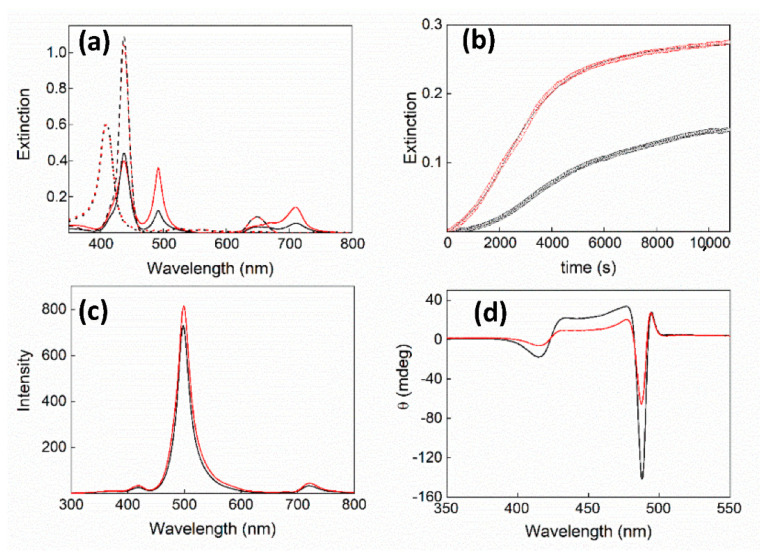
(**a**) Extinction spectra before (dotted line), soon after initial mixing (dashed lines), and at the end of the aggregation (solid lines), (**b**) kinetic extinction time traces at λ = 492 nm (solid lines show the best fit of the experimental data according to Equation (1)), (**c**) RLS, and (**d**) CD spectra for the supramolecular assembling of H_2_TPPS_4_^4−^ induced by salt and tartrate buffer (L: black lines; D: red lines) using mix 2 protocol. Experimental conditions: [H_2_TPPS_4_^4−^] = 3 μM; [NaCl] = 1.5 M; tartrate buffer 100 mM, pH 2.7, T = 298 K.

**Figure 6:**
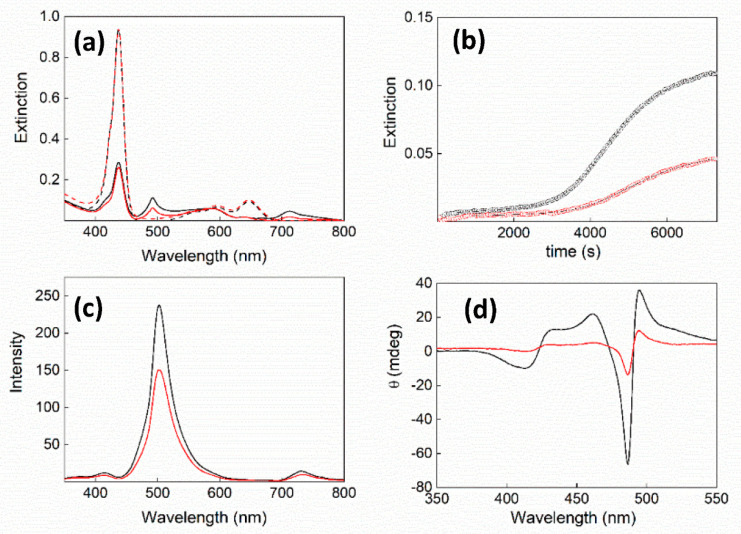
(**a**) Extinction spectra soon after initial mixing (dashed lines) and at the end of the aggregation (solid lines), (**b**) kinetic extinction time traces at λ = 492 nm (solid lines show the best fit of the experimental data according to Equation (1)), (**c**) RLS, and (**d**) CD spectra for the supramolecular assembling of H_2_TPPS_4_^4−^ induced by salt and tartrate buffer (L: black lines; D: red lines) using mix 3 protocol. Experimental conditions: [H_2_TPPS_4_^4−^] = 3 μM; [NaCl] = 1.5 M; tartrate buffer 100 mM, pH 2.7, T = 298 K.

Previous investigations showed that enantiomeric forms of tartaric acid and the corresponding anion are able to imprint chirality in growing J-aggregates of H_2_TPPS_4_^4−^, imposing a specific handedness to the supramolecular structures [24,34,35,65]. An important finding was that L- and D-tartrate exhibited a strong difference in the aggregation rates, with the L isomer being much faster than the D one. In addition, the final dissymmetry g-factor for the J-aggregates, related to the quality of the CD oscillator, was different, favoring the L enantiomer with respect to D [35]. Therefore, this particular system has been chosen to investigate the role of the mixing order when a fourth component, i.e., the chiral anion, is used to access specific supramolecular assemblies having selected properties such as chirality. The approach adopted in this case is quite similar to the above-mentioned one, since we added the chiral species as tartrate buffer, thus implying a premixing of acid (H^+^) and the chiral tartrate anion. Therefore, the mixing methodologies consist in (i) adding tartrate buffer to a pre-diluted porphyrin solution, and subsequently adding NaCl (mix 1); (ii) adding NaCl to a pre-diluted porphyrin solution and then the tartrate buffer (mix 2); (iii) adding a premixed solution of NaCl and tartrate buffer to a pre-diluted porphyrin solution (mix 3) (for details, see the Experimental Section). All the kinetic traces have been analyzed according to Equation (1) and the relevant parameters are reported in Table 2. A comparative analysis of the data between the two enantiomers for the various mixing protocols reveals that the values of the rate constants *k_c_* are not very different, except for mix 1, while the average value of *m* is three (the only exception being the L-enantiomer with mix 2), and *n* is comprised in the range 2–7, very close to other literature data [27]. Another interesting observation is that the rate constant *k_c_* values decrease in the order mix 1 > mix 2 ≈ mix 3, only in the case of the L-enantiomer. This trend is similar to that found for the aggregation in the absence of tartrate buffer (Table 1). Figure 4, Figure 5 and Figure 6 report a collection of the relevant spectroscopic data for the different mixing protocols.

When the tartrate buffer is added to the pre-diluted porphyrin solution, the initial extinction spectrum corresponds to the diacid species (mix 1, Figure 4a), even if a slight bathochromic shift (Δλ = +4 nm) is evident. This shift is also detectable in applying the other mixing protocols, pointing to some ion pairing between the porphyrin monomer and the tartrate or alternatively, as suggested by a reviewer of the manuscript, the formation of a porphyrin dimer mediated by tartrate anion. The subsequent addition of salt triggers the self-assembling process with formation of J-aggregates. As already pointed out in the literature [35], both the kinetic rate constants and the amount of aggregated porphyrins are different for the two enantiomers, L being faster and more efficient than D-tartrate in promoting the process. This experimental evidence has been tentatively attributed to the intermediacy of highly reactive oligomeric species able to discriminate between the two enantiomeric forms of tartrate in the rate-determining step [35]. The extinction at the end of the kinetics (Figure 4a) and the corresponding RLS profiles at 492 nm (Figure 4c) are more intense in the case of the L-enantiomer. The kinetic time traces exhibit the usual sigmoidal profiles (Figure 4b) with quite different lag-times. In the case of L-tartrate, while the catalyzed pathway is almost three times faster than the other enantiomer, the uncatalyzed pathway *k*_0_ makes a more important contribution to the overall process, being almost 50-fold larger with respect to D-tartrate. This shows a remarkable difference from the other two mixing protocols relays in relation to the sign of the Cotton effect in the exciton CD spectra that are negative for L and positive for D (Figure 4d). In addition, the intensity of the CD bands for the L-enantiomer is larger than that of the D-enantiomer. These findings are in agreement with the kinetic chiral discrimination reported in previous investigations on these J-aggregates [35]. When salt is added to the pre-diluted porphyrin solution (mix 2), as pointed out above, the initial species is an H-dimer, as clearly evidenced by the hypochromicity and hypsochromic shift (Δλ = −4 nm) of the Soret band (Figure 5a, dotted line). The latter converts into the oligomeric species upon addition of the tartrate buffer (Figure 5a). In this case, an inversion of the rate constant *k_c_* for the catalyzed pathway was observed for the two enantiomers, together with an unusually high value for the number of porphyrin units in the initial nucleus (m ≈ 11) for L (Table 2). Different from the mix 1 protocol, in this case the CD spectra of the final J-aggregates, even if different in intensity, exhibit the same sign (Figure 5d). A similar result is the general outcome of the experiments performed adopting mix 3 protocol. J-aggregates show a chirality independent from the handedness of the chiral inducer (Figure 6d). On these bases, we can reasonably assume that only when the chiral anion A^−^ (Scheme 2, mix 1) interacts initially with the diacid species H_4_TPPS_4_^2−^ is it able to imprint its handedness on the growing supramolecular system. The initial nucleus should be a porphyrin trimer; a P- or M-helical chirality could be imprinted at this level and amplified in the subsequent auto-catalytic growth. When mix 2 or 3 protocols are used, the presence of high concentrations of sodium cations suppresses the interaction between the porphyrin monomer or oligomers and the chiral inducer, thus leading to a positive Cotton effect. Indeed, CD spectra with positive couplets are those usually found in literature in the case of spontaneous symmetry breaking for these J-aggregates [39,66].

## 3. Materials and Methods

### 3.1. Materials

The sodium salt of 5, 10, 15, 20-*tetrakis*(4-sulfonatophenyl)porphyrin (Na_4_TPPS_4_) was purchased from Aldrich (Milan, Italy). NaCl, high purity D- (99%, optical purity ee 97%) and L-tartaric acid (≥99.5%, optical purity ee 99%) were purchased from Sigma. The pH of the tartrate buffer (100 mM) was adjusted to 2.7 by adding NaOH to the solution of tartaric acid. The HCl was of the highest commercial grade available and was used as received without further purification from Fluka. All the aqueous solutions were prepared in high-purity doubly distilled water (HPLC grade, Fluka, Milan, Italy). A stock solution of porphyrin (200 μM) was freshly prepared and stored in the dark. The concentration used in the experiments was calculated by UV/Vis absorption spectroscopy using the molar extinction coefficients at the B-band (H_2_TPPS_4_^4−^: 5.33 × 10^5^ M^−1^cm^−1^, λ = 414 nm).

### 3.2. Methods

UV/Vis extinction spectra were acquired on an Agilent 8453 diode array spectrophotometer. A UV filter (Hoya glass type UV-34, cut-off: 340 nm) was always set in the light path between the lamp and the measurement cell to avoid any photo-damage of porphyrin samples, especially during kinetic experiments. Kinetic runs were executed by acquiring spectra from cells placed in the thermostatic sample holder of the spectrophotometer (298 K). All the reactions were started rigorously following three different reagent mixing order protocols: (i) addition of a proper volume of salt stock solution to a pre-acidified diluted porphyrin sample (mix 1), (ii) addition of a proper volume of acid stock solution to a diluted porphyrin sample pre-mixed with salt (mix 2), (iii) addition of a proper volume of a pre-acidified stock solution of salt to a diluted porphyrin sample (mix 3). For all samples, the final porphyrin concentration has been fixed at 3 μM. All the stocks solutions were rigorously thermostated and then added at the same temperature with respect to the sample holder (298 K).

In the case of kinetic experiments triggered in presence of HCl (0.3 M), the concentration of NaCl has been fixed to 0.2 M and all the kinetic runs were carried out using a conventional spectrophotometric quartz cell (1 cm path length). In a typical mix 1, 30 μL of concentrated stock solution of porphyrin (200 μM) were added to high-purity doubly distilled water for obtaining a diluted solution of porphyrin directly in the cell. The following step consisted in the addition of 200 μL of concentrated stock solution of acid (3 M). Then aggregation was triggered by addition of 200 μL of concentrated stock solution of NaCl (2 M) after equilibration of the pre-acidified porphyrin solution (about 2 min). As regards to the other mixing protocols (mix 2 and 3), keeping fixed the concentration of reagents as indicated in advance, they exclusively differ for the order of their addition as specified above.

For aggregation studies conducted in presence of tartrate buffer (100 mM pH 2.7), the final concentration of NaCl has been fixed to 1.5 M and all the kinetic experiments were carried out using a special, tightly closed tandem cuvette (Hellma Tandem Cell, 238-QS, Milan, Italy). The volumes of the solutions were kept equal for each chamber and held separately until the aggregation was triggered. In a typical mix 1, 30 μL of concentrated stock solution of porphyrin (200 μM) were added to tartrate buffer (200 mM pH 2.7) aqueous solution in one of the two chambers of the spectrophotometric quartz cell (0.5 cm path length). The other chamber was filled with an equal volume of NaCl aqueous solution (3 M). In a typical mix 2, 30 μL of concentrated stock solution of porphyrin (200 μM) were added to NaCl aqueous solution (3 M) in one of the two chambers of the cell, whereas the other one was filled with an equal volume of tartrate buffer (200 mM pH 2.7). In a typical mix 3, 30 μL of concentrated stock solution of porphyrin (200 μM) were added to high-purity doubly distilled water in one of the two chambers of the cell, whereas the other one contained an equal volume of an aqueous solution of NaCl (3 M) in tartrate buffer (200 mM pH 2.7). For all the mixing order protocols, after equilibration of the solutions present in the two chambers, a rapid mix of the two volumes started aggregation.

The experimental extinction data versus time collected at 492 nm were analyzed by a non-linear least square fit to the equation:

Ext_t_ = Ext_∞_ + (Ext_0_ − Ext_∞_) (1 + (*m* − 1){*k*_0_t + (*n* + 1)^−1^ (*k_c_*t)*^n^*^+1^})^−1/(*m*−1)^ (1)

where Ext_0_, Ext_∞_, *k*_0_, *k_c_*, *m*, and *n* are the parameters to be optimized [63].

CD spectra were measured on a Jasco J-710 spectropolarimeter. Resonance light scattering (RLS) spectra were acquired on a Jasco FP-750 spectrofluorimeter, adopting a synchronous scanning protocol of both excitation and emission monochromators and a right angle geometry [67]. Time resolved fluorescence emission measurements were done on a Jobin Yvon-Spex Fluoromax 4 spectrofluorimeter using Time—Correlated Single-Photon Counting technique. A NanoLED (λ = 390 nm) was used as excitation source.

## 4. Concluding Remarks

Supramolecular systems could exhibit hierarchical structures with a high level of complexity. Therefore, a precise knowledge of all the thermodynamic and kinetic parameters involved in their growth processes is required in order to access specific features such as expression and control of chirality. The possibility of transferring, amplifying, and memorizing chirality in these nano-assemblies represents a fascinating subject [68]. J-aggregates of sulfonatophenylporphyrins are simply obtained under acidic environments, but depending both on the experimental conditions and mixing protocols, a plethora of different structural motifs can be obtained. The role of the order of adding the various components into the solution has been clearly demonstrated. The protocols can strongly affect the nature of the initial species and their interaction with eventual other components. A precise procedure is required to obtain supramolecular systems were chirality is imprinted, reflecting the handedness of the chiral templating reagent.

## Figures and Tables

**Table 1:** Relevant kinetic parameters for the J-aggregation of H_2_TPPS_4_^4−^ (*k*_0_, *k_c_*, *m*, and *n*) as functions of different mixing order protocols. Parameters were developed by adopting a porphyrin first (PF) mixing protocol by using HCl at different concentrations.

	10^5^ × *k*_0_/s^−1^	10^4^ × *k_c_*/s^−1^	*m*	*n*
Mix 1	7.61 ± 0.24	10.30 ± 0.01	3.2 ± 0.1	3.5 ± 0.1
Mix 2	1.93 ± 0.21	5.27 ± 0.01	4.1 ± 0.1	7.8 ± 0.2
Mix 3	0.90 ± 0.18	3.85 ± 0.01	3.0 ± 0.1	7.6 ± 0.2
PF-HCl ^a^	0.78 ± 0.03	1.95 ± 0.01	3.7 ± 0.1	7.4. ± 0.2
PF-HCl ^b^ [27]	22.01 ± 0.04	8.91 ± 0.02	2.4 ± 0.1	4.8 ± 0.1

[H_2_TPPS_4_^4−^] = 3 μM, [HCl] = 0.3 M, [NaCl] = 0.2 M, T = 298 K at 492 nm. ^a^ [HCl] = 0.3 M. ^b^ [HCl] = 0.5 M. Kinetic data from non-linear fit of extinction data to Equation (1).

**Table 2:** Relevant kinetic parameters for the J-aggregation of H_2_TPPS_4_^4−^ (*k*_0_, *k_c_*, *m*, and *n*) as functions of different mixing order protocols.

	10^5^ × *k*_0_/s^−1^	10^4^ × *k_c_*/s^−1^	*m*	*n*
		**L**		
Mix 1	20.2 ± 0.1	10.2 ± 0.1	3.1 ± 0.1	1.8 ± 0.1
Mix 2	26.1 ± 0.2	3.9 ± 0.1	10.8 ± 0.9	4.0 ± 0.2
Mix 3	1.9 ± 0.1	2.9 ± 0.1	2.8 ± 0.1	6.8 ± 0.1
		**D**		
Mix 1	0.4 ± 0.1	3.8 ± 0.5	2.5 ± 0.1	3.6 ± 0.1
Mix 2	12.3 ± 1.1	5.1 ± 0.1	2.6 ± 0.1	2.6 ± 0.2
Mix 3	1.7 ± 0.2	2.9 ± 0.01	2.8 ± 0.4	6.6 ± 0.4

[H_2_TPPS_4_^4−^] = 3 μM, tartrate buffer 100 mM pH = 2.7; [NaCl] = 1.5 M, T = 298 K from non-linear fit of extinction data to Equation (1) at 492 nm.
